# Dissociable Spatial and Temporal Effects of Inhibition of Return

**DOI:** 10.1371/journal.pone.0044290

**Published:** 2012-08-31

**Authors:** Zhiguo Wang, Jan Theeuwes

**Affiliations:** Department of Cognitive Psychology, Vrije Universiteit, Amsterdam, The Netherlands; University of Groningen, The Netherlands

## Abstract

Inhibition of return (IOR) refers to the relative suppression of processing at locations that have recently been attended. It is frequently explored using a spatial cueing paradigm and is characterized by slower responses to cued than to uncued locations. The current study investigates the impact of IOR on overt visual orienting involving saccadic eye movements. Using a spatial cueing paradigm, our experiments have demonstrated that at a cue-target onset asynchrony (CTOA) of 400 ms saccades to the vicinity of cued locations are not only delayed (temporal cost) but also biased away (spatial effect). Both of these effects are basically no longer present at a CTOA of 1200 ms. At a shorter 200 ms CTOA, the spatial effect becomes stronger while the temporal cost is replaced by a temporal benefit. These findings suggest that IOR has a spatial effect that is dissociable from its temporal effect. Simulations using a neural field model of the superior colliculus (SC) revealed that a theory relying on short-term depression (STD) of the input pathway can explain most, but not all, temporal and spatial effects of IOR.

## Introduction

To quickly adapt to an ever changing environment, an organism must practice efficient visual orienting. Because new objects or events may convey important information, orienting is reflexively drawn towards new objects when they appear in a scene [1]. Once an object has been attended, the visual system is subsequently biased against sampling the same spatial location, resulting in less efficient processing of objects appearing there. This bias is labeled inhibition of return (IOR) [2] and has been regarded as a mechanism that encourages orienting towards novelty [3]–[7].

### The Neural Underpinning of IOR

IOR is typically explored with a spatial cueing paradigm in which a target is preceded by an uninformative cue [8]. The target can appear at a cued location or an uncued, distance matched, location. Shortly after cue presentation, responses to targets appearing at the cued location are usually faster than to those appearing at uncued locations. This facilitation effect is generally attributed to capture of attention by the onset of the cue [2], [3], [8]. When the cue-target onset asynchrony (CTOA) exceeds 200 ms, however, a behavioral cost (IOR) emerges at the cued location: responses to targets appearing at cued locations are delayed compared to those appearing at uncued locations [3] (see [9] for review). As suggested by Posner and Cohen [3], one possible explanation of IOR is that “Some part of the pathway from the cued location may be reduced in efficiency by the cueing” (p. 537). This hypothesis is echoed by recent theorization of IOR as habituation [10], repetition suppression [11], onset detection cost [12], and short-term depression (STD) of early sensory input [13].

The STD theory of IOR proposed by Satel and colleagues [13] is intuitive and computationally explicit (see also [14]). Due to cue-elicited STD, visual input strength is reduced for targets that appear in the neighborhood of the cued location, leading to slower response times (RTs). The dynamics of target input reduction can be described as a function of CTOA with an alpha function [13]:
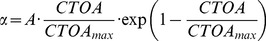
(1)where *A* is the maximum amount of input reduction and *CTOA_max_* is the CTOA at which the reduction reaches its maximum. This input based theory is backed by single-unit recordings in the superior colliculus (SC), a midbrain structure that contains a topographic motor map encoding the vector of saccadic eye movements (for a recent review, see [15]). Dorris and colleagues [16] showed that behavioral IOR is accompanied by a reduction of neuronal response in the intermediate layers of the SC (SCi). Electrical stimulation of the SCi [16] and recording in the superficial layers of the SC (SCs) [17] suggest that this reduction in neuronal response originates from upstream visual pathways. This finding is consistent with human EEG (see [18] for a review) and fMRI [19] studies, which have shown that behavioral IOR is accompanied by reduced activation in the visual cortex. With simulations in a neural field model of the SC [20], Satel and colleagues [13] demonstrated that the STD theory can explain a variety of neurophysiological and behavioral observations in the IOR literature.

It is important to note that, as implied in Equation 1, STD (and thus IOR) starts with the presentation of the cue. According to Satel and colleagues [13], the early facilitation effect is usually observed in cueing paradigms because the residual activation of the cue is stronger than the STD (see also [10], [12], [21]).

### Spatial Effect of IOR

In their seminal IOR paper, Posner and Cohen [3] proposed that IOR evolved to “reduce the effectiveness of a previously active area of space in summoning attention” and thus to “maximize the sampling of the visual environment” (p. 550). Although this functional explanation of IOR is now widely accepted in the field [4], [5], [22]–[25], the implied spatial effect of IOR on visual orienting responses (e.g., reaching and eye movements) has not been fully explored (but see [26]–[28]).

Posner, Rafal, Choate and Vaughan [2] were the first to report that eyes are less likely to go to a cued than to an uncued location. However, a lack of measurement of the prototypical temporal cost of IOR makes it difficult to infer whether this spatial effect is caused by IOR. This methodological limitation has been remedied in recent studies of oculomotor search. For instance, Klein and MacInnes [7] observed that, in a difficult search task, saccade latencies were longer to probes presented at previously fixated locations than to those presented at locations that were not yet fixated (temporal cost of IOR). Suggesting the existence of a spatial effect of IOR, Klein and MacInnes [7] found that a larger portion of saccades were directed away from previously fixated (and thus attended) locations ( see also [29]–[32]).

When two proximal visual stimuli are presented simultaneously, a first saccade is usually directed to an intermediate location [33]–[35]. This phenomenon has been referred to as the “global effect” [33]. Watanabe [28] explored whether IOR interacts with the global effect by combining a spatial cueing paradigm [8] with a double target paradigm [33]. Participants were required to make a saccade to either one target or one of two spatially proximal targets. On trials in which only one target was presented, slower saccadic response times (SRTs) were observed when a cue was presented at the target location 600 ms before target onset (revealing temporal IOR). On trials with double targets, many saccades landed near the mid-point between the targets when no cue was presented (the global effect). When one of the paired targets was cued, however, the average landing positions of saccades drifted away from the cued location, suggesting that IOR can bias averaging saccades away from cued locations. In a second experiment, Watanabe [28] showed that the relationship between IOR and the global effect could also be observed when a visual target was accompanied by a visual distractor (see also [26]).

### Purpose of the Present Study

As discussed previously, the literature seems to suggest that, in addition to the well-established temporal effect (see [36] for a graphical meta-analysis), IOR may also have a spatial effect on overt orienting responses (e.g., saccades). That is, IOR biases saccades away from previously attended locations. However, due to several methodological and theoretical issues, the spatial effect of IOR needs further empirical exploration. First, it is clear that attention can be captured reflexively by external events (exogenous shift), or can be controlled voluntarily by an organism’s internal intentions (endogenous shift) [37]. In the classic cueing task with which IOR was first demonstrated and has been frequently explored since, IOR is believed to be caused by an exogenous shift of attention [2]; in an oculomotor search task in which the participant pursues the goal of finding a predetermined target, however, IOR is caused by endogenous shift of attention. Thus, caution should be taken when generalizing findings of the oculomotor search literature to the spatial cueing literature. Second, the neural mechanism of the global effect is still unclear [38]. It is difficult to determine whether the findings of Watanabe [28] can be regarded as unambiguous evidence for a spatial effect of IOR. Third, in the spatial cueing task, the magnitude of behavioral IOR changes as a function of CTOA [36]. However, it is unclear whether the spatial effect of IOR, if it exists, also varies with CTOA. Fourth, it is unclear whether the STD theory of IOR, which has been shown capable of capturing the temporal effect of IOR observed in monkeys, can also accommodate a spatial effect of IOR.

To determine the time course of the spatial effect of IOR, the present study used a modified cueing task in which the CTOA was 200 ms, 400 ms or 1200 ms. Consistent with the notion of both a temporal and spatial effect of IOR on the oculomotor system, at a CTOA of 400 ms, saccades to the vicinity of the cued location were not only delayed (temporal effect) but also biased away (spatial effect). Both of these effects were largely absent at a CTOA of 1200 ms. When a 200 ms CTOA was tested, a temporal benefit (i.e., faster responses to targets appeared in the vicinity of the cued location) was observed, replicating the prototypical finding of the cueing paradigm [3], [36]; however, saccades still robustly deviated away from the cued locations. These findings suggest that IOR has dissociable temporal and spatial effects. Simulations using a neural field model of the SC further demonstrated that the STD theory of IOR [13] can accommodate most but not all the observed spatial and temporal effects of IOR.

## Results

**Figure 1:**
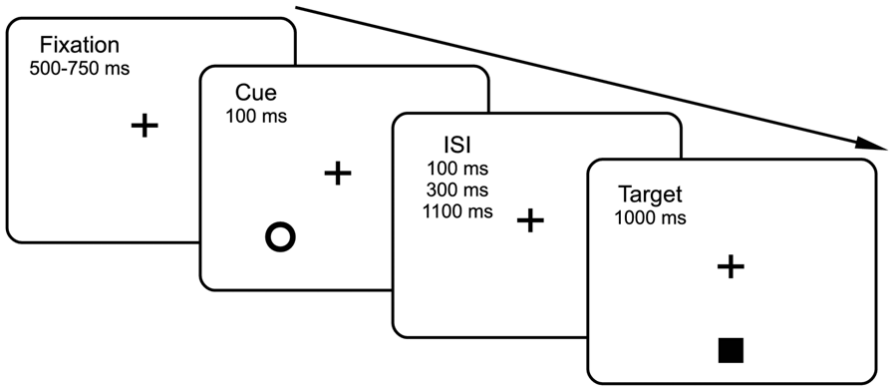
Sequence of events in a sample trial. The stimuli are not drawn to scale. In this illustrated experimental trial the cue is presented 45° away from the target, although, as described in the text, cues could appear 15°, 30°, 45°, 60°, 75°, 90°, 105°, 120°, 135°, 150°, or 165° (degree of arc) from the target, either in the left or the right visual field. The CTOA was set to 200 ms, 400 ms and 1200 ms for three separate groups of participants.

To explore the spatial effect of IOR, we used a modified cueing task in which the target appeared at the same eccentricity as the cue but its angular distance to the cue was varied between 15°-165° (see Figure 1 for an illustration). Because previous studies have shown that the temporal effect of IOR reaches its maximum at a CTOA of about 400 ms [36], we first tested a 400 ms CTOA. This experiment revealed a robust spatial effect of IOR, that is, the initial saccade direction deviated away from the cued location. To characterize the time course of this spatial effect, we further tested short (200 ms) and long (1200 ms) CTOAs with separate groups of participants. Furthermore, to verify whether the findings of these behavioral experiments can be accommodated with the STD theory of IOR [13], we performed simulations in a neural field model of the SC [13], [20], [39].

### Behavioral Experiments

**Figure 2:**
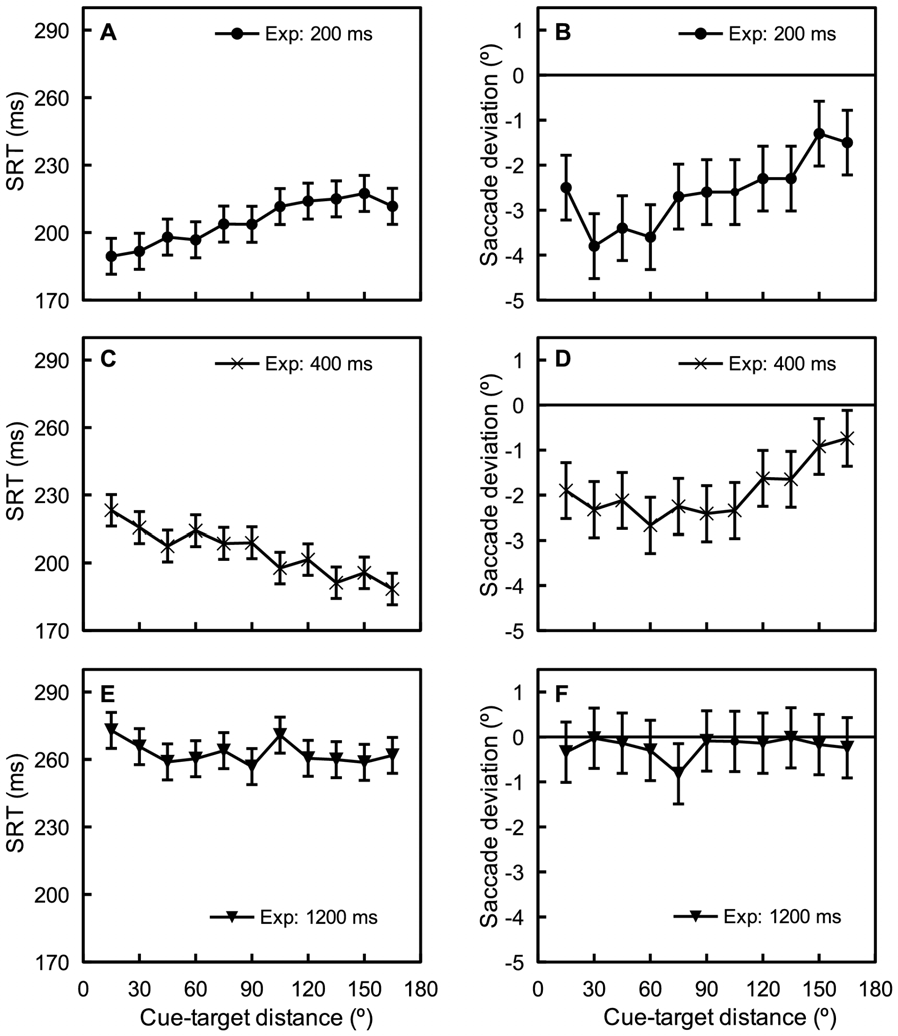
Mean SRTs (A, C, E) and saccade deviations (B, D, F) for each cue-target-distance of the three CTOAs. Error bars denote 95% within-subject confidence intervals based on the error term of cue-target distance [50].

The task procedure is illustrated in Figure 1. The target was presented either above or below fixation along the vertical axis, while the cue could appear either in the left or right visual field (see Methods and Materials). Participants were instructed to ignore the cue and to quickly saccade to the target (if presented). The velocity threshold used to determine whether a saccadic eye movement was made was set to 35°/s, and saccadic response times (SRTs) were computed by subtracting the time at which the eye movement exceeded the velocity threshold from the time at which the target appeared on screen. Deviation in initial saccade direction relative to the target direction was used to index the spatial effect of IOR. This measurement was signed, with positive and negative values denoting deviation towards and away from the cued visual field, respectively. Initial saccade direction was estimated with the starting point of a saccade and the gaze position 10 ms into the same saccade (for similar measures of saccade direction, see [40], [41]). The mean SRTs and saccade deviations are presented in Figure 2.

### Saccadic response time (SRT)

An ANOVA performed on the SRTs, with variables cue-target distance (15°, 30°, 45°, 60°, 75°, 90°, 105°, 120°, 135°, 150°, or 165°) and CTOA (200 ms, 400 ms, or 1200 ms), revealed a significant main effect of CTOA, F(2, 33)  =  11.66, p < 0.001, η_G_^2^  =  0.38 (generalized eta squared [42], [43]). This main effect occurred because the overall SRT for the 1200 ms CTOA was about 60 ms longer than those for the 200 ms and 400 ms CTOAs. The main effect of cue-target distance was not significant, F(10, 330)  =  1.17, p  =  0.31, η_G_^2^  =  0.00, however, the interaction between cue-target distance and CTOA did reach significance, F(20, 330)  =  8.06, p < 0.001, η_G_^2^  =  0.05. This interaction occurred because SRT increased with cue-target distance at the 200 ms CTOA, F(10, 110)  =  6.08, p < 0.001, η_G_^2^  =  0.08, but decreased with cue-target distance at the 400 ms CTOA, F(10, 110)  =  10.32, p < 0.001, η_G_^2^  =  0.07, and the 1200 ms CTOA, F(10, 110)  =  1.83, p  =  0.06, η_G_^2^  =  0.02. This pattern of results replicates the classic time course of IOR observed in human subjects, that is, response times to targets presented at (or close to) the cued location are shortened at relatively short CTOAs and are prolonged when the CTOA exceeds about 250 ms [3], [36].

### Saccade deviation

An ANOVA of saccade deviations, with variables cue-target distance and CTOA, also revealed a significant main effect of CTOA, F(2, 33)  =  11.06, p < 0.001, η_G_^2^  =  0.28. As is clear from Figure 2, saccades deviated away from the cued visual field and the amount of deviation decreased with CTOA. The main effect of cue-target distance was significant, F(10, 330)  =  5.54, p < 0.001, η_G_^2^  =  0.07, suggesting that the amount of deviation was modulated by cue-target distance. A significant interaction between cue-target distance and CTOA was also observed, F(20, 330)  =  1.85, p < 0.05, η_G_^2^  =  0.05, suggesting that this modulation effect differed across the CTOAs. Though evident at the 200 ms CTOA, F(10, 110)  =  4.50, p < 0.001, η_G_^2^  =  0.13, and at the 400 ms CTOA, F(10, 110)  =  3.94, p < 0.001, η_G_^2^  =  0.10, the modulation of saccade deviation by cue-target distance was largely absent at the 1200 ms CTOA, F(10, 110)  =  0.55, p  =  0.85, η_G_^2^  =  0.04.

### Simulations

**Figure 3:**
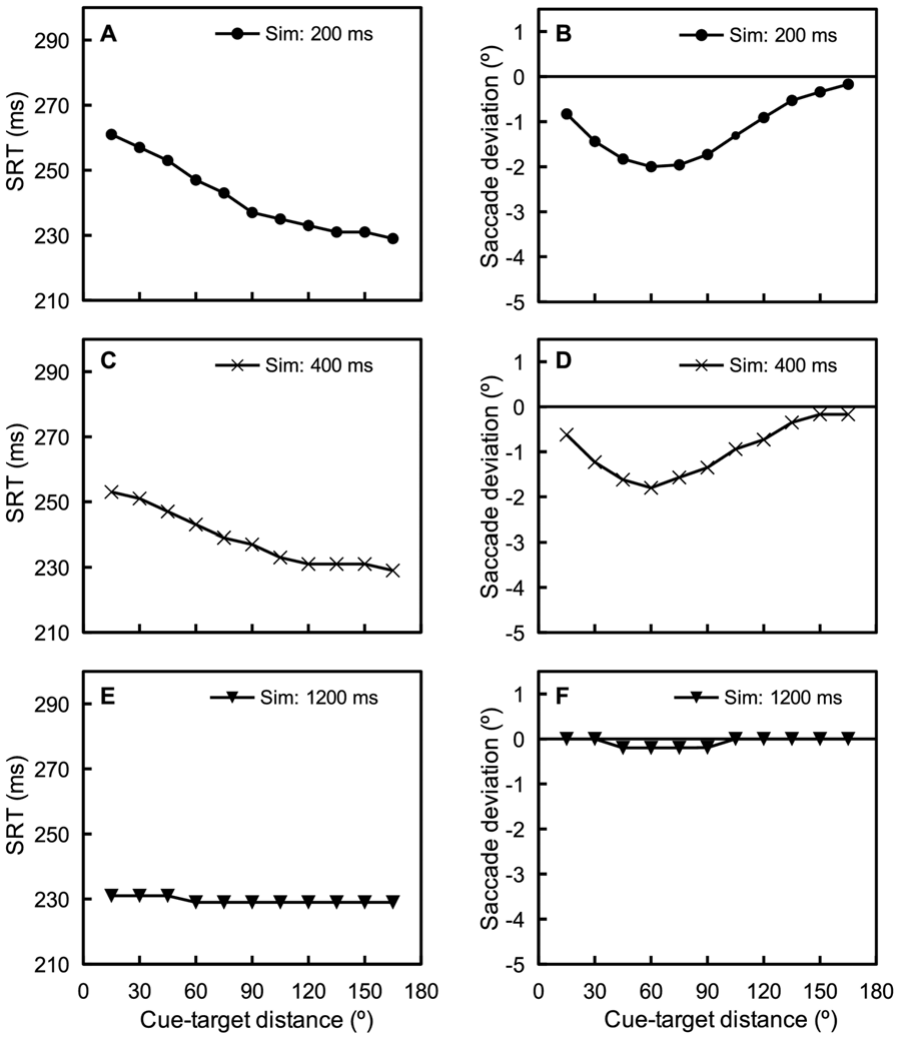
Simulated SRTs (A, C, E) and saccade deviations (B, D, F) for each cue-target-distance of the three CTOAs.

As shown in Figure 2, the present experiments revealed that the temporal and spatial effects of IOR have different time courses. To determine whether the STD theory of IOR [13] can accommodate these observations, simulations were performed using a neural field model of the SC [13], [20], [39]. The architecture and parameters of this model have been documented in detail in other places [13] (see Methods and Materials). The simulated SRTs and saccade deviations are presented in Figure 3.

For saccade deviations (i.e., the spatial effect of IOR), the simulations successfully reproduced the pattern of results obtained in the present experiments. Saccade deviations decreased with CTOA, with the strongest deviation observed in the 200 ms CTOA simulation and the weakest observed in the 1200 ms CTOA simulation. It is important to note that the modulation effect of cue-target distance on saccade deviation was elegantly captured by the model. As in behavioral experiments, nonlinear relationships between cue-target distance and saccade deviation were obtained in both the 200 ms and 400 ms CTOA simulations. For the 1200 ms CTOA, the simulations produced fairly weak but noticeable deviations when the cue-target distance was between 45°-90°. Interestingly, a small amount of statistically reliable deviation was also observed in the corresponding behavioral experiment when the cue-target distance was 75°, t(11)  =  2.97, p < 0.05.

The principle behind our successful simulation of the spatial effect of IOR is straightforward. As has been shown in previous behavioral studies, IOR has a spatial gradient centered around the cued location [44], [45]. When the target is presented close to the cued location, the exogenous target input is unevenly attenuated by the cue-elicited STD, with the side closer to the cued location being attenuated more than the other side. As a result, the peak of the target evoked activation is biased away from the cued location, leading to saccade deviation away from cues. The further the distance to the cued location the weaker the bias. This is why deviation away from the cued location was relatively weak when the target was distal to the cued location at both the 200 ms and 400 ms CTOAs.

For SRTs, the model reproduced the pattern of behavioral results of the 400 ms and 1200 ms CTOAs, but failed to capture the results of the 200 ms CTOA experiment. The SRTs increased with cue-target distance in the 200 ms CTOA experiment, however, in corresponding model modulations, SRTs decreased as the cue-target distance increased. This failure of the model was no surprise because, using the same STD parameters, Satel and colleagues [13] also produced a similar IOR effect at a 200 ms CTOA. Contrary to the popular belief that IOR takes about 200 ms to appear, the STD theory of IOR states that STD (and thus IOR) starts with the cue (see also [3], [9]). Early facilitation effects are often observed in cueing paradigms because the effect of the STD is overshadowed by residual activation from the cue at very short CTOAs. In the model, however, the cue-related activation is largely diminished by 200 ms after cue onset, leaving only STD affecting SRTs. Thus, the model cannot produce a facilitation effect at relatively long CTOAs (e.g., 200 ms) without further assumptions or components.

It should be noted that the simulated SRTs were faster for the 1200 ms CTOA than for the 200 ms and 400 ms CTOAs, while the behavioral experiments produced the opposite pattern of results. This is because temporal expectation of target appearance, which is reflected by the well-known warning signal or foreperiod effect [46]–[48], was not considered in our simulations.

## Discussion

The present experiments explored whether IOR has a spatial effect and how this spatial effect (if present) develops over time. To this end, we used a modified cueing task in which not only the CTOA, but also the cue-target angular distance, were systematically varied. Replicating previous observations in spatial cueing paradigms [3], [36], a facilitation effect was observed at a short (200 ms) CTOA and IOR effects were observed at relatively long (400 ms and 1200 ms) CTOAs ( see Figure 2). Importantly, saccades to targets were found to deviate away from the cued location, suggesting that IOR also has a spatial effect. The strength of this spatial effect decayed with time, and was modulated by cue-target angular distance (see Figure 2). In addition, to determine whether the mathematically explicit STD theory of IOR [13] can accommodate these findings, simulations were performed with a neural field model of the SC [13], [39]. These simulations elegantly reproduced the observed spatial effect of IOR, but failed to reproduce the temporal facilitation effect observed at the 200 ms CTOA.

### Spatial Effects of IOR

In addition to the widely recognized temporal effect [36], the present experiments revealed that IOR also has a spatial effect, that is, IOR biases saccades away from cued locations. We want to point out that we are not the first to report a spatial effect of IOR. As has been discussed before, several previous studies have suggested that IOR affects the spatial properties of oculomotor responses. For instance, previous studies have found that the eyes are less likely to land at previous fixated locations where IOR-like temporal costs are observed [7], [29]–[32], and averaging saccades in response to spatially proximal stimuli are repelled by exogenous cues [26], [28], [49]. One unique contribution of the present experiments is that we show that the spatial IOR effect evoked by exogenous cueing systematically varies with the cue-target distance. As clearly shown in Figure 2 (error bars denote 95% within-subject confidence intervals [50]), this relationship is nonlinear: the strongest deviation was not observed for cues presented closest to the target. This is in contrast to the temporal effect of IOR which is strongest when cues are presented closest to the target or at the same location as the target [44], [45].

Another important finding of the present experiments is that the spatial and temporal effects of IOR are dissociable, the strongest spatial effect was observed at the 200 ms CTOA where a temporal facilitation effect, rather than IOR, was observed. Using endogenous targets (i.e., arrows at fixation), a similar dissociation between temporal and spatial effects of exogenous cueing was reported in Theeuwes and Godijn [51]. In a recent replication of Watanabe's study [49], we also found that averaging saccades were consistently repelled by cues regardless of the presence or absence of temporal IOR. We have no satisfying explanation for such dissociations. It is possible that top-down factors, such as temporal expectation [49] and attentional control settings [52], have asymmetric modulatory effect on the temporal and spatial dynamics of oculomotor processing (see also [30], [51]). Future empirical investigation of this issue is strongly encouraged.

### Causes of Saccade Deviation

Previous studies have shown that saccades may deviate towards or away from task irrelevant visual distractors [53]–[55]. The dominant theory in the literature attributes such distractor evoked deviations to active inhibition or suppression of the distractor location unfolding over time: early in time when the suppression is weak deviation towards is observed, while later in time when the suppression is fully developed deviation away appears [27], [41], [56]. If this theory is correct, one would predict that neuronal activation in the SC would be reduced when deviation away from distractors is observed. To test this prediction, in a recent cell-recording study White, Theeuwes and Munoz [57] tested a 400 ms stimulus onset asynchrony (SOA) so as to allow for enough time for suppression to buildup at the distractor location. They found that the distractor evoked neuronal activation in the SC did not differ when saccades deviated towards or away from distractors. This finding led us to propose that distractor evoked saccade deviation is not caused by suppression of distractor-related activation but rather by the lateral interaction structure of the SC [58], which is characterized by short-distance excitation and long-distance inhibition (for a summary of relevant evidence, see [59], [60]). This theory predicts that saccades deviate towards close distractors and deviate away from distal distractors (see [61] for similar ideas and supporting behavioral data). However, the lateral interaction in the SC could not be the main cause of saccade deviations observed in the present experiments because, at all CTOAs, no deviation towards cued locations was observed, even when the cue-target distance was only 15° (angular distance).

As has been discussed previously, IOR can cause saccade deviations because it exerts an uneven inhibition on target-related activation in the SC. Similarly uneven inhibition can be achieved by pharmacologically inactivating an SC site (see [62], [63]). In either case, saccades would be biased away, even if the target is presented very close to the affected area (i.e., the cued location or the response field of the inactivated SC site). The difference is that inactivation directly suppresses activation within the SC while IOR probably suppresses activation in the upstream input pathway.

### Limitations of the STD Theory of IOR

Since the STD theory of IOR, by its very nature, is an input-based theory, it is important to acknowledge that this theory cannot explain all findings in the IOR literature (see also [64]). First, slower saccadic eye movements to previous fixated locations has been labeled as IOR [7], [29], although this observation is not caused by STD in the visual pathway but rather by the internal dynamics of the SC [39], [64]. Second, many scholars have used endogenous targets (e.g., arrows at fixation) to reveal the effects of IOR generated by exogenous cueing [51], [65], [66]. Because endogenous targets are not presented in the visual pathway previously stimulated by exogenous cues, behavioral IOR effects observed in this class of studies cannot be handled by the STD theory. Third, the STD process, as has been suggested by Satel and colleagues [13], is restricted to the early visual pathway and thus operates on retinotopic coordinates. This theory cannot handle previous observations of spatiotopic [3], [44] and object-based coding [67] of IOR. Fourth, previous studies have shown that IOR effects appear later in time when perceptually demanding processing (i.e., target discrimination) is required than when simple detection or localization responses are required (see [12] for a review). Since the STD theory considers only the effect of STD on target visual input, it cannot explain this observation without further assumptions. Finally, as has been demonstrated in the present simulations, the STD theory cannot generate temporal facilitation effects at relatively long CTOAs (e.g., 200 ms). This finding seems to suggest that, in addition to the residual activation of the cue, there are other factors contributing to the early facilitation effects observed in cueing paradigms (see [12]). This issue needs to be addressed in future studies.

### Conclusions

The present experiments show that, in a cueing task, saccades can be biased away from the cued location. This spatial effect decays with time and is dissociable from the classic temporal effects observed in cueing tasks (i.e., shortly following the presentation of the cue, responses to targets appearing at the cued location are facilitated, while later in time this temporal benefit is replaced by a temporal cost). Simulations with a neural field model of the SC suggest that the STD theory of IOR [13] can explain the spatial effects observed in the present experiments, but cannot explain the temporal facilitation effect observed at a 200 ms CTOA. Regardless of the underlying mechanism(s) of IOR, the present observation of saccade deviation following exogenous cueing is in agreement with the supposition that IOR biases orienting toward novel locations [3]–[5].

## Materials and Methods

The human research protocol described here was approved by the Ethical Committee of Vrije Universiteit and all participants gave written informed consent.

### Participants

All participants (N =  37) of the present study reported normal, or corrected-to-normal, vision. Except for the first author (ZW) who participated the 400 ms CTOA experiment, all participants were compensated with money (9 Euro per hour) or course credits. One participant from the 1200 ms CTOA experiment was dropped from analysis because she finished less than 50% of the trials. The average age of the remaining 36 participants (18 female and 18 male) was 21 years. The CTOAs (200 ms, 400 ms or 1200 ms) were manipulated between-subjects, with each CTOA tested with 12 participants.

### Stimuli, Apparatus and Procedure

All stimuli were drawn in gray on a black background and were presented on a 21 inch CRT monitor. Stimulus presentation and timing of events were controlled by custom software written in Python. A video-based Eyelink^®^ eye tracker (SR Research), with a spatial resolution of 0.2° or better, was used to record the gaze direction of the participants at a sampling rate of 500 Hz.

The cue was an empty circle (d  =  0.8°) and the target (the imperative stimulus) was a filled square (0.8°×0.8°). The sequence of events in a sample experimental trial is illustrated in Figure 1. To ensure an accurate reading of gaze position, self-paced drift correction was performed at the beginning of each trial. Following the drift correction, a gray fixation cross (0.8°×0.8°) appeared at the center of the display for 500–750 ms. Then, the cue was presented for 100 ms. After an inter stimulus interval (ISI) of 100 ms, 300 ms or 1100 ms, the target appeared 8° above or below the central fixation cross. To discourage participants from using the cue as a warning signal, the cue was not presented on 20% of the trials. When presented, the cue could appear at a location 15°, 30°, 45°, 60°, 75°, 90°, 105°, 120°, 135°, 150°, or 165° (degree of arc) from the target, either in the left or the right visual field. To discourage anticipatory responses, the target was not presented on 20% of the trials, regardless of the presentation of the cue. Warning messages were displayed if participants failed to maintain fixation before target onset or the primary saccade missed the target by more than 4° (visual angle). After a practice block of 24 trials, the participant was tested with 2 blocks of 275 trials. To ensure an accurate recording of the gaze coordinates, a 9-point calibration procedure was administered at the beginning of each block or whenever a break was required by the participant.

### Behavioral Data Analyses

Trials were discarded if: a) the participant failed to maintain fixation before target onset (5.1%, 4.9% and 11.6% for the 200 ms, 400 ms and 1200 ms CTOA experiments, respectively), b) the primary saccade went to the hemifield opposite to the target (2.7%, 1.6% and 0.4%), c) the landing position of the primary saccade missed the target by more than 4° (5.7, 4.4% and 3.0%), d) the SRT was faster than 100 ms or slower than 500 ms (6.2%, 4.7% and 4.1%), or e) the primary saccade deviated more than 30° (degree of arc) from the target direction (3.7%, 2.3% and 4.1%). After this data cleansing procedure, 84.9%, 87.5% and 81.6% of non-catch trials were left for the 200 ms, 400 ms and 1200 ms CTOA experiments, respectively. ANOVAs were then performed on the SRTs and saccade deviations, with cue-target distance (15°, 30°, 45°, 60°, 75°, 90°, 105°, 120°, 135°, 150°, or 165°) as a within-subjects factor and CTOA (200, 400 or 1200 ms) as a between-subjects factor.

### Model Architecture

The neural field model used in the present study has been documented in detail in previous works by Trappenberg and colleagues [13], [20], [39]. This is a 1-dimensional model which represents only the direction of saccades. Nodes in this model are leaky integrators and the dynamics of the internal state *u_i_(t)* of a node is described by Equation 2, with a time constant of *τ  =  10.* A sigmoid gain function was used to relate the average firing rate *r_i_(t)* of a node to its internal state (Equation 3), with a steepness of *β  =  0.07.*

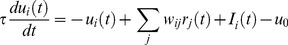
(2)

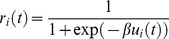
(3)


Previous studies have shown that neurons in SCi are laterally connected in a manner such that proximal neurons excite each other and distal neurons inhibit each other [68], [69]. The connection strength *w_ij_* between two nodes *i* and *j* is defined in Equation 4.
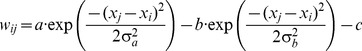
(4)


As in previous work [13], [20], [70], the external input to a node was dissected into exogenous input (*visual signal*) and endogenous input (*a hypothetical “go” signal from higher cortical areas*). Both exogenous and endogenous inputs were assumed to have a Gaussian spatial shape [59] (see Equation 5).
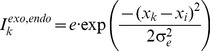
(5)


### Model Parameters

#### Architectural parameters

N  =  1000 nodes were used to represent 5 mm of SCi tissue. The lateral connection parameters were the same as those used by Trappenberg and colleagues [13], [20], [39], *a  =  72, b  =  24, c  =  6.4*, *σ_a_  =  0.6 mm*, *σ_b_  =  1.8 mm*. These parameters were optimized to approximate cell recordings in the monkey SC [20].

#### Input parameters

The width of both exogenous and endogenous inputs were estimated to be *σ_exo,endo_  =  0.7 mm* [20]. The strength of exogenous and endogenous inputs were set to *e_exo_  =  55* and *e_endo_  =  12,* respectively. The exogenous input decayed exponentially (with a time constant of 10), while the endogenous inputs were sustained until saccade onset [13], [39].

#### Output parameters

25 ms after the activity level of a node reached 80% of its maximum firing rate, a saccade into its response field was assumed to be initiated. A scale factor of 2 and an additional afferent delay of 70 ms [71] were used to convert simulation cycles to behavioral SRTs.

#### STD (IOR) parameters

Cell recordings in the monkey SCs suggest that the reduction in neuronal response to cued visual targets reaches its maximum (26%) at a CTOA of about 100 ms [17]. No such neurophysiological data is available for humans. For easy comparison to previous work, the present simulations used the same STD parameters used by Satel and colleagues [13], *A  =  −0.49* and *CTOA_max_  =  220 ms*. Furthermore, we assumed that STD has a Gaussian shaped spatial gradient, centered at the cued location [45]. The amount of STD at node *i*, depending on its distance to the cued node (*k*), is described in Equation 6. The width of the STD gradient (*σ_std_*) is largely unknown. Because a previous behavioral study by Bennett and Pratt [45], which measured manual RTs to targets presented at various distance from the cued location, suggests that IOR has a fairly large spatial gradient and affects RTs throughout the visual field, a width of *σ_std_  =  1.4 mm* was used in our simulations. It should be noted that the purpose of our simulations was to determine whether the STD theory of IOR can accommodate the pattern of our behavioral results, rather than to fit our behavioral data. The pattern of our simulation results can also be produced with a set of slightly different parameters.
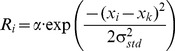
(6)

